# A roadmap for a Metaverse-based digital governance: A case of the Gambia

**DOI:** 10.1371/journal.pone.0314388

**Published:** 2024-12-02

**Authors:** Pa Sulay Jobe, Murat Yilmaz, Hüseyin Emre Ilgın

**Affiliations:** 1 Informatics Institute, Gazi University, Ankara, Turkey; 2 Department of Computer Engineering, Gazi University, Ankara, Turkey; 3 School of Architecture, Faculty of Built Environment, Tampere University, Tampere, Finland; Guangdong University of Petrochemical Technology, CHINA

## Abstract

This paper explores the integration of Metaverse technologies into the digital governance framework of The Gambia, in order to identify the sectors where these innovations can be implemented most effectively. Driven by the pursuit of cutting-edge solutions to improve governance, improve public service delivery, and promote citizen engagement, the study uses qualitative analysis methodologies, including interviews with Information Technology (IT) professionals and an examination of government reports. Through the application of NVivo software for data analysis, essential themes and areas suitable for Metaverse applications were uncovered. These include the potential of the Metaverse to fundamentally transform sectors such as education, public service, infrastructure development, urban planning, and cultural heritage tourism. Despite recognizing the capacity of the Metaverse for transformative impact, this research identifies significant obstacles, including technical infrastructure readiness, ethical issues, and the need for policy reform. The findings highlight the need to address these challenges through substantial improvements in digital infrastructure, literacy, ethical guidelines, and policy frameworks. The study calls for a multistakeholder collaboration model, which incorporates government, the private sector, academia, and international bodies, to ensure the successful integration of the Metaverse into the digital governance of The Gambia. Offering comprehensive insights and strategic recommendations, this research underscores the collective effort required of all stakeholders to harness the full potential of the Metaverse, thus facilitating an inclusive and efficient digital governance ecosystem in the Gambia.

## Introduction

In the evolving landscape of technological innovation, pivotal advances such as the Internet and smartphones have redefined the societal structures and governance frameworks. Among these technologies, Metaverse stands out as a revolutionary concept that promises a new chapter in digital governance. The Metaverse can be defined as an expansive digital space that exceeds typical online interactions, providing immersive environments for diverse activities, effectively merging professional and social experiences [1].

Digital governance has witnessed significant milestones, transitioning from paper-based bureaucracies to e-government platforms that revolutionized public administration through digital means. This shift, driven by the advent of Internet technologies, has streamlined administrative processes and broadened avenues for civic engagement [2]. As governance embraced the digital era, it used information and communication technology (ICT) to improve the delivery of public services, increase civic participation, and improve government operational efficiency [3]. The evolution from informational to transactional and interactional models in digital governance marked a pivotal shift toward more inclusive and efficient public services, driven by the use of strategic ICT to facilitate government functions [4].

During the global transition to digital governance, Gambia has embarked on an ambitious path to harness digital technologies for public sector modernization. Based on the vision articulated by the Estonian Academy of e-Governance [5] and detailed in the Gambia Digital Economy Master Plan, the nation aspires to implement more than 500 public digital services. This initiative extends beyond infrastructure improvements and is integral to a broader strategy to develop a digital ecosystem conducive to innovation and entrepreneurship. Collaborating with educational institutions and technology companies to establish digital hubs aims to fortify ICT and digital education capabilities, preparing the workforce for forthcoming technological shifts, including the Metaverse [6]. The proactive stance of Gambia in adopting ICT for public administration positions it as a model for the African continent, reflecting a commitment to leveraging digital advances for service delivery and governance transparency. The nation’s digital governance efforts, as laid out in its master plan, highlight the importance of fostering public-private partnerships, which are crucial to driving digital transformation and innovation and ensuring inclusivity in the digital governance landscape [6].

Despite the numerous opportunities presented by digitalization in governance, it also presents considerable challenges. Issues such as the digital divide, privacy, security, and equitable access to resources present potential hurdles to seamlessly integrating technologies such as the Metaverse [7]. However, the capacity of digital governance to increase efficiency, transparency, and citizen participation illustrates the vital role of technology in strengthening democratic processes. As The Gambia pushes ahead in its digital governance journey, it faces the dual challenge of navigating these impediments while seizing opportunities to reimagine public administration and citizen engagement through the lens of the Metaverse.

The Metaverse, with its promise of an expansive virtual domain, introduces new avenues for reimagining governance through immersive digital environments. This platform can transcend conventional e-government systems, enabling more dynamic and comprehensive public administration and service delivery modes. As nations around the world, including Barbados with its establishment of a digital embassy and China and Korea with significant investments in Metaverse initiatives [8], pivot toward integrating Metaverse into their governance models, The Gambia is likely to benefit from this emerging trend. The implications of the Metaverse extend beyond governance, touching sectors such as education, healthcare and urban development, thus magnifying its utility as a tool for societal advancement [9].

### Research aim and objective

Guided by the question:

*What sectors within The Gambia’s digital governance framework are identified by technology personnel as most conducive to integrating Metaverse technologies?*

This study identifies the primary areas within the Gambia’s digital governance framework for the Metaverse technology application, focusing on insights from the nation’s IT professionals. The objectives are structured as follows.

- To evaluate the landscape of digital governance in the Gambia.
- To investigate the potential applications of immersive technologies within this framework.
- Assess both the technological and social readiness for such integration.
- To identify prevailing challenges and concerns and develop practical recommendations for incorporating Metaverse technologies.

This paper adopts a thematic qualitative research approach to explore the essential sectors within the Gambia’s digital governance that are ripe for Metaverse technology integration. Interviews with IT professionals from various sectors in the Gambia provide the empirical foundation, while government reports and academic research offer a comprehensive framework for analysis. The analysis facilitated by NVivo, reveals themes and patterns from the data, paving the way for an in-depth understanding of Metaverse integration opportunities within The Gambia’s digital governance framework.

## Literature review

Digital governance involves the strategic deployment of digital technologies in government functions, with the aim of fostering a more efficient, transparent, and participatory public administration system [10]. This approach encompasses managing a nation’s digital assets, services, and data to enhance service delivery and citizen engagement. Beyond mere digitization of services, digital governance involves the comprehensive integration of information and communication technologies (ICT) into governance frameworks, catering to the evolving needs of modern societies.

The importance of digital governance in modern public administration cannot be overstated. In an era of rapid technological advancements and changing citizen expectations, governments face mounting pressure to adapt and innovate. This requires effective frameworks for managing these changes, transforming how services are delivered, how citizens interact with the government, and how information is managed and used for public benefit [11].

Examples of implementation of digital governance include Estonia’s eGovernance model, which streamlines governmental processes through innovations such as e-residency and electronic voting [12], Singapore’s Smart Nation initiative, which uses ICT to improve healthcare, security and urban living [13], and India’s Aadhaar project, which provides a biometric digital identity to more than a billion citizens, reducing fraud and increasing efficiency [14]. Digital governance also promotes transparency through open data and e-participation platforms, fostering trust between governments and citizens, a cornerstone of democratic governance [15].

Furthermore, digital governance impacts citizen engagement by using digital tools to solicit feedback, co-create services, and encourage community involvement in governance processes. This participatory approach improves the relevance and quality of public services, while empowering citizens and fostering a sense of ownership and responsibility towards community development and governance [16].

### Digital governance in Africa

Africa’s digital governance landscape presents a contrast to experiences in regions like Europe, primarily due to resource constraints, digital literacy gaps, and underdeveloped or developing infrastructure. Despite these challenges, the continent is experiencing a digital revolution, with governments increasingly turning to digital technologies to enhance service delivery, transparency, and citizen participation [17].

Strategic investments in digital infrastructure are bridging the digital divide, streamlining administrative processes, reducing corruption, and improving service delivery [18, 19]. For example, South Africa and Nigeria have effectively used digital health technologies during the COVID-19 pandemic, improving disease surveillance and disseminating health information [20]. In addition, Kenya’s ICT innovations in agriculture have empowered farmers with real-time information on weather patterns, pest infestations, and crop prices, contributing to food security [21].

In Ghana, digital solutions have transformed water management, introducing smart meters and management systems that monitor usage and detect leaks, enhancing water conservation and the quality of public utilities [22]. Rwanda’s Irembo platform represents a significant step toward digital governance, offering a one-stop-shop portal for more than 100 services, streamlining citizen access to various government services, and reducing opportunities for corruption [23].

However, achieving comprehensive digital governance in Africa requires interdisciplinary partnerships, collaborative strategies, and meticulous execution, ensuring successful implementation and improving citizen engagement. Persistent challenges include digital divides, cybersecurity concerns, and infrastructure deficits, particularly in urban centers, as seen in Ghana and other regions of Africa [24, 25]. A conducive socio-economic environment that supports the adoption and growth of technologies is necessary for these strategies to succeed [26].

### Digital governance in the Gambia

The Gambia, with a population exceeding 2.4 million inhabitants, faces unique challenges and opportunities in its digital governance landscape. Approximately 57% of its population resides in urban areas, highlighting the importance of urban-centric digital initiatives [27]. The country’s economy is based on a diverse mix of industries, including tourism, agriculture, and construction, with emerging growth in the services and manufacturing sectors [5]). This economic diversity underscores the importance of adopting a comprehensive digital strategy to propel the Gambia’s development trajectory.

**Fig 1:**
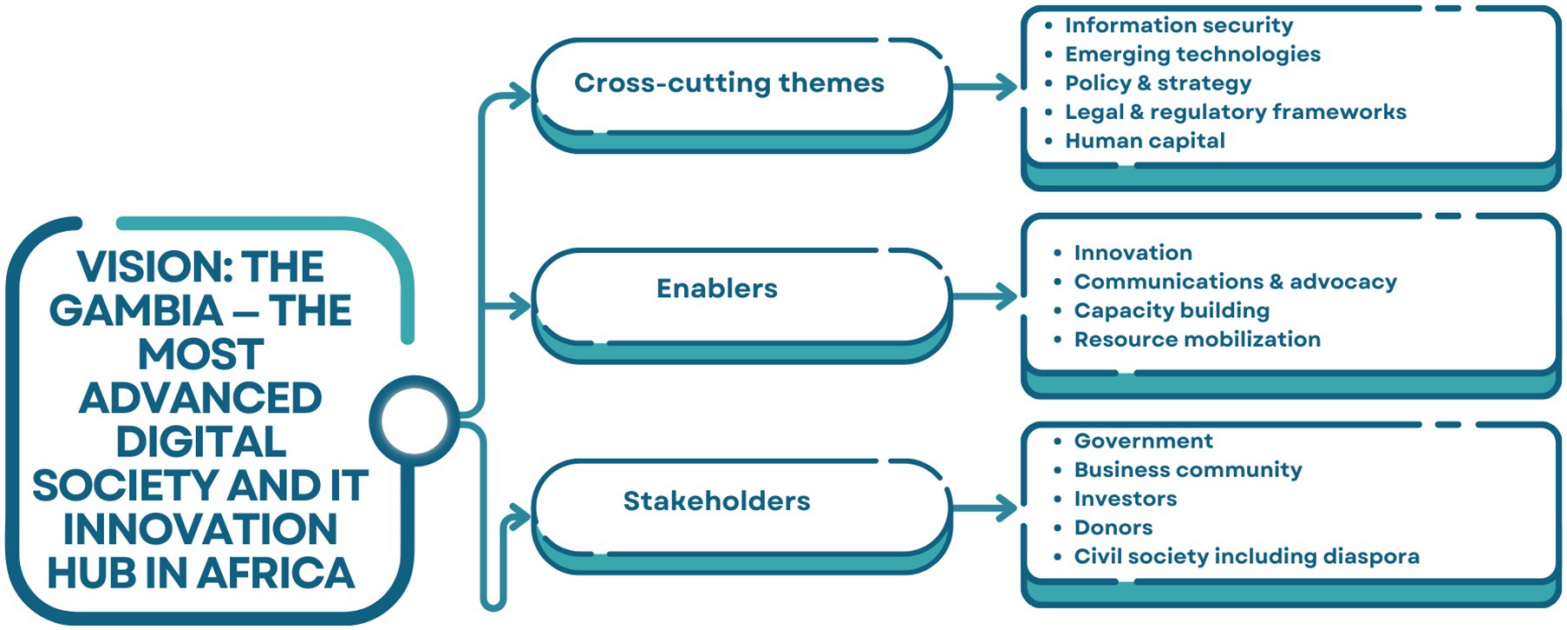
The Gambia digital economy framework by e-governance academy of Estonia [5].

*The Gambia Digital Economy Diagnostic* report by Muller et al. [27] provides a detailed assessment of the country’s digital governance framework, identifying areas of progress and potential for further development (see Fig 1) The country aims to leverage digital technologies across public sectors to stimulate economic growth and improve the efficiency of public services. Key focus areas include improving digital literacy, expanding financial inclusion, and advancing the digitalization of government services. These initiatives are crucial to foster inclusive growth and ensure that the benefits of digital transformation reach all segments of society.

In line with this vision, The Gambia has outlined a forward-looking strategy through its *Digital Economy Master Plan 2023-2033*, envisioning a society empowered by robust digital infrastructure, comprehensive cybersecurity measures, and digitized government operations to drive sustainable economic and social development. The plan emphasizes multi-stakeholder collaboration, with partnerships between the government, private sector, and international allies playing a pivotal role in realizing the Gambia’s digital aspirations. The plan addresses challenges such as infrastructure enhancement, workforce upskilling, and regulatory reforms, prioritizing inclusivity and resilience in shaping the country’s digital future.

The COVID-19 pandemic accelerated the adoption of digital governance practices in Gambia, highlighting the resilience of its governance model in times of crisis [28]. Central to the success of these initiatives is the Technology Acceptance Model (TAM), which shows that perceived usability and value will significantly influence the adoption of e-government services [29, 30]. The Gambia’s efforts to introduce online tax filing systems, business digital registration services, and healthcare appointment digital platforms have yielded positive results, streamlined processes, and improved accessibility of services.

Table 1 outlines some statistical indicators of The Gambia.

**Table 1:** Statistical indicators about The Gambia (adopted from [5]).

Indicator	Value
Population	approx. 2.5 million
Total area	10,689 square kilometers
Urban/peri-urban habitation	More than 50%
GDP per capita in 2021^1^	835.6 USD
Annual growth of GDP per capita in 2021^2^	5.6%
Unemployment rate in 2021^3^	11.2%
Access to electricity (of the population)^4^	62%
Use of the Internet (of the population)^5^	37%
Individuals owning mobile phones (of the population)^6^	81%
Literacy rate^7^	51%
Active mobile subscribers in 2021^8^ (2020^9^)	2.7 million (2,637,032)
Active Internet subscriptions in 2020^10^	1,833,452

While The Gambia faces certain challenges in the digital infrastructure, progress is being made toward improving connectivity and access. According to the Gambia Digital Economy Diagnostic [27], the penetration of mobile broadband is steadily growing, with 4G services now reaching 4.5% of the population, and more than 60% of the population is covered by a mobile broadband signal. However, the high cost of broadband services and limited accessibility remain areas of improvement, which the government is addressing through the Digital Economy Master Plan [5]. These infrastructure upgrades will play a crucial role in supporting the integration of Metaverse technologies and broader digital transformation efforts.

The diagnostic highlights that The Gambia has significant potential for increased digital engagement. The ownership of mobile phones is high, with 93% of households owning a mobile device, signaling a strong foundation for future digital services. Although the current usage of mobile money is 2%, efforts are underway to expand financial literacy and access to digital financial services. In addition, initiatives to strengthen e-Government services will improve the global standing of Gambia, as the country continues to build on its National Development Plan and digital strategies. These ongoing efforts create a promising environment for the adoption of advanced technologies like the Metaverse.

### The Metaverse

The Metaverse, a term first coined by Neal Stephenson in his 1992 novel *Snow Crash*, describes a virtual reality space where users, through avatars, engage within a fully immersive computer-generated environment [31]. Originally a concept of speculative fiction, Metaverse has evolved into a multifaceted digital ecosystem that integrates cutting-edge technologies such as virtual reality (VR), Augmented reality (AR), and blockchain [32]. This transformation marks a significant shift in digital interaction and connectivity.

The word *Metaverse* combines meta (meaning beyond) and universe, suggesting a realm that extends beyond physical boundaries to forge a comprehensive and interactive digital world [33]. Initially the domain of gaming and entertainment, the scope of the Metaverse has broadened significantly, catalyzed by advances in VR and AR technologies, which enable highly sophisticated simulations of physical reality and immersive digital experiences. VR technology immerses users in virtual environments that mimic real-world sensations, while AR technology blends digital components into the physical view, improving daily interactions with layered information [34].

The adoption of Metaverse into mainstream digital ecosystems gained momentum when Facebook (now Meta) announced its strategic pivot toward building a comprehensive Metaverse, signaling a societal embrace of virtual spaces as the next frontier of digital engagement [35]. Blockchain technology has introduced more levels of security, transparency and decentralization in the Metaverse, supporting a robust digital economy where virtual assets translate into real-world value, facilitating secure and transparent transactions [36].

The literature on the Metaverse has expanded significantly in recent years, with numerous studies exploring its applications and challenges in various domains, including cultural heritage, training, and education.

Buragohain et al. [37] examine the challenges, opportunities, and strategies involved in digitalizing cultural heritage through Metaverse applications. Their study identifies critical obstacles, such as technological limitations and concerns about data privacy. It also highlights the transformative potential of immersive experiences to improve cultural heritage preservation and education, outlines strategic recommendations to leverage Metaverse technology to engage with cultural assets effectively, and emphasizes the balance between innovation and preservation needs.

Yamyuan et al. [38] explore the pioneering implementation of Metaverse technologies in education and industry in ASEAN countries. Their study highlights how virtual environments are transforming teaching and learning paradigms, as well as enhancing industrial collaboration on an international scale. The insights from their research provide valuable lessons for The Gambia, demonstrating the potential benefits and strategies for successful Metaverse integration in education and industry sectors.

Buragohain et al. [39] present a systematic study on the impact of immersive learning on teacher effectiveness. The research evaluates how immersive learning technologies improve teacher training and professional development, and ultimately improve educational outcomes. The findings suggest that experiential and immersive environments significantly contribute to teacher performance by providing innovative ways to engage with learning materials and pedagogical practices.

Buragohain et al. [40] analyze the potential impact and prospects of the Metaverse in educational settings through a combination of systematic reviews and case study research. Their findings highlight both the benefits and challenges of incorporating immersive technologies in learning environments, particularly in response to educational disruptions caused by the COVID-19 pandemic. The study emphasizes that Metaverse applications can enhance learning experiences. However, it also underscores the urgent need to address technological and infrastructural challenges to maximize effectiveness, making the audience feel the importance and urgency of research.

Yilmaz et al. [41] explore the potential of Metaverse technologies in training and educational environments, specifically focusing on the SAFe framework. The study presents empirical results showing how immersive virtual environments can improve learning outcomes and participant participation. The findings suggest that Metaverse-based training offers significant benefits over traditional methods, particularly in scalability and experiential learning, opening up exciting possibilities for the future of education.

### Opportunities for the Metaverse in governance

The integration of the metaverse into governance signifies a shift, introducing innovative avenues for public service delivery, citizen engagement, and redefinition of government operations. In its essence, Metaverse merges the creative potential of art with functional advancements of ICT, creating a vibrant space where user-generated content and social networks thrive [42]. This platform extends beyond technological innovation to improve citizen participation and design of digital services, allowing participation design and collaborative decision-making [43].

The Metaverse’s capacity to transform digital connectivity includes creating virtual laboratories for remote learning and training, and enhancing professional development. Virtual settings allow for the testing of policies and response strategies in controlled, yet realistic environments, improving preparedness and response effectiveness [44]. The Metaverse’s role as a design space for developing governance solutions involves leveraging new technologies and creative ideas, driving the digital evolution of public services [45].

Recognizing the potential of the digital economy, the Gambian government has taken proactive steps to embrace digital transformation. Initiatives such as the introduction of digital electronic birth certificates, the Civil Service e-Recruitment Portal, and the Automated System for Customs Data (ASYCUDA), among others, illustrate significant progress in the migration from analog to digital service delivery at the national level [27]. Building on these foundational services, the Ministry of Communications and Digital Economy has outlined strategic plans that aim to enhance network connectivity, improve regulatory policies, and strengthen e-government services, including cybersecurity, in partnership with the University of the Gambia to promote innovation in information technology and digital solutions.

**Fig 2:**
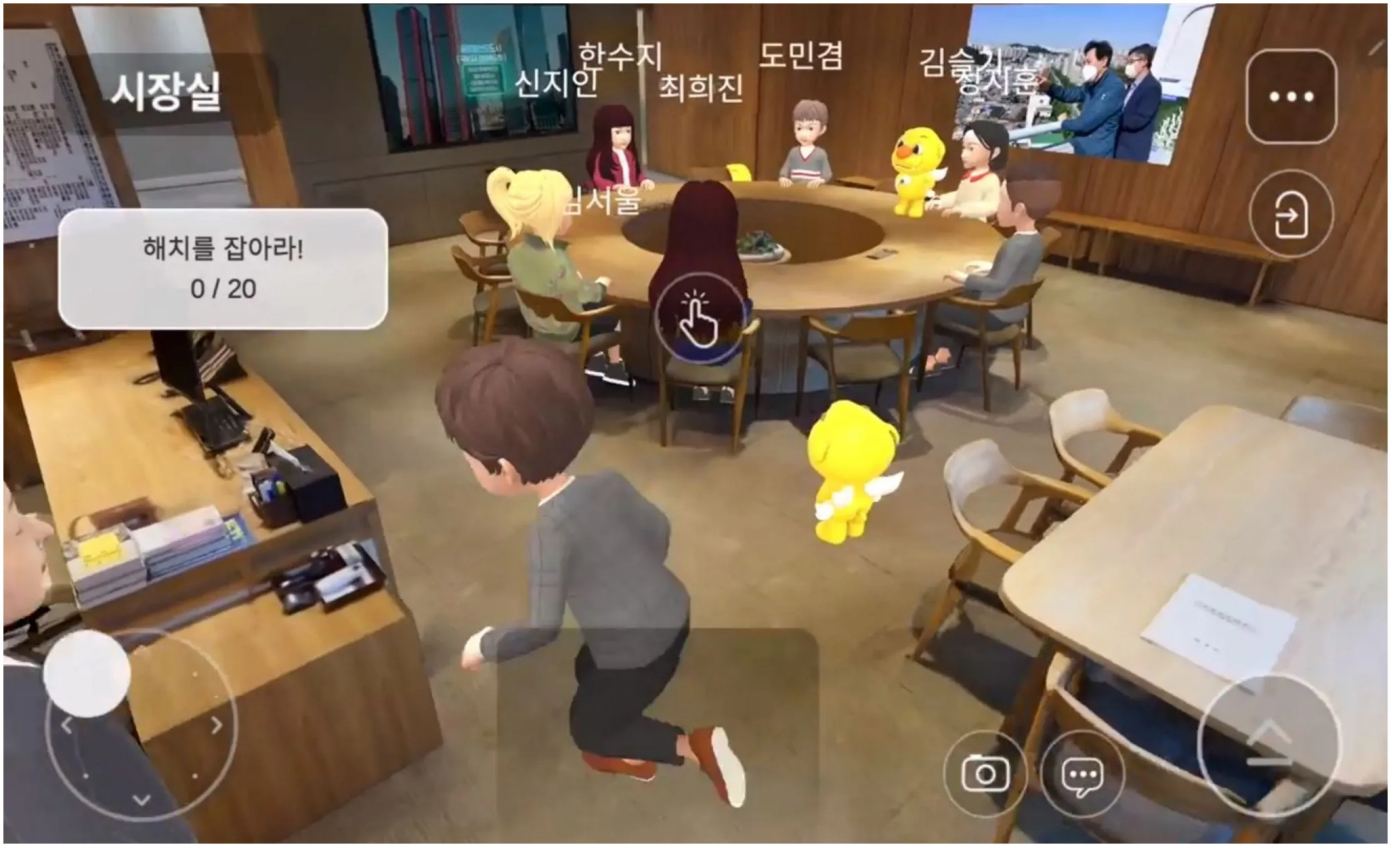
The Metaverse Seoul [46].

Using VR and AR technologies, the Gambia can improve the delivery of public services, deepen citizen engagement, and strengthen the integrity of digital transactions. The Metaverse can also transform education by creating dynamic three-dimensional environments, enhancing learning methodologies [47]. Models like Metaverse Seoul can inspire The Gambia to develop virtual platforms for public services, streamlined service provision, and accessibility (see Fig 2) [48].

In healthcare, the Metaverse can revolutionize patient care and service delivery through immersive educational experiences, enhancing treatment understanding and outcomes [49]. Tourism can also benefit from immersive technologies, providing virtual tours that enrich pre-visit experiences, promote the preservation of cultural heritage, and expand educational opportunities [50]. The Gambia can leverage these technologies to create engaging virtual tours of its landmarks, extending its reach to tourism.

### Challenges and considerations in integrating the Metaverse

**Fig 3:**
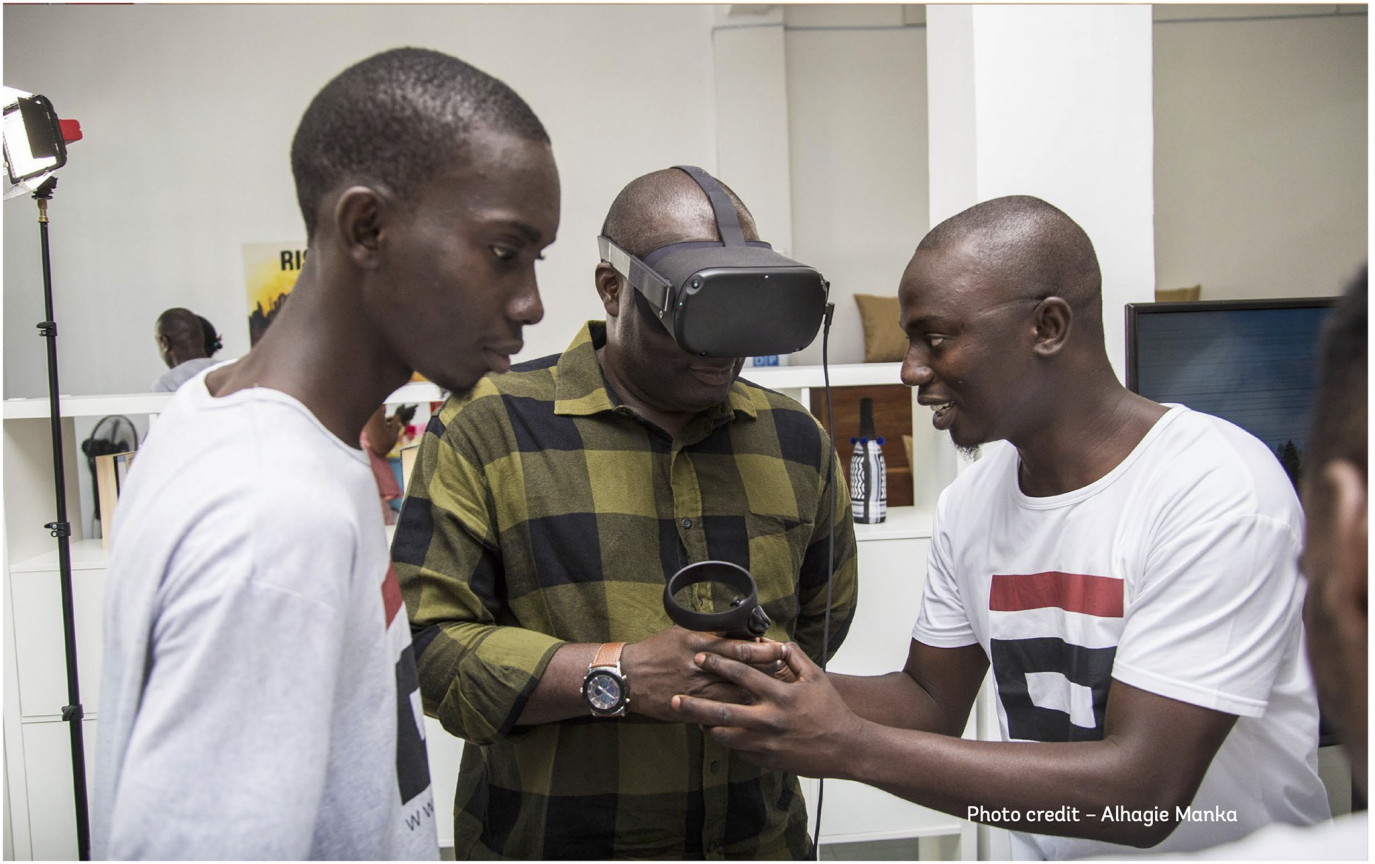
Technology exhibition in The Gambia [27] (Adopted from The Gambia digital economy diagnostic, page 111).

Integrating the Metaverse into governance brings about significant challenges (see Fig 3). Establishing a technological infrastructure is crucial to support data-intensive applications, as outlined in the Gambia’s “Digital Economy Master Plan 2023-2033”. Infrastructure gaps persist, particularly in rural areas, limiting access to Metaverse applications and risking socioeconomic inequalities [27].

The interconnected nature of Metaverse raises privacy and security concerns, which require tight cybersecurity measures to safeguard user information [51]. The governance of virtual spaces requires adaptive and forward-thinking regulatory frameworks, learning from initiatives such as the European Union’s General Data Protection Regulation (GDPR) to balance innovation and protection [52].

To overcome these challenges, the Gambia must prioritize investments in digital infrastructure, broadband connectivity, and digital literacy programs [53]. Cost barriers must also be addressed, using solutions such as Google Cardboard for accessible VR experiences [54]. Government subsidies and public access points with technology resources can democratize access to the Metaverse’s benefits.

## Methodology

This study adopts a qualitative research design, utilizing semi-structured interviews to explore how Metaverse technologies can be integrated into the Gambia’s digital governance. The choice of a qualitative approach allows for a comprehensive understanding of the perspectives of IT professionals on the potential and challenges of Metaverse technologies within governance structures [55]. In addition, the study incorporates secondary data from government reports, providing objective metrics on the readiness of the Gambia’s digital infrastructure and technology adoption rates.

### Sampling strategy

A purposive sampling strategy, complemented by snowball sampling, was employed to identify and select Information Technology (IT) professionals with specific knowledge or experience relevant to digital governance and Metaverse technologies. Purposive sampling ensured that the collected data were rich and directly applicable to the research questions [56]. The selection criteria included IT personnel in the Gambia who have knowledge or experience with the Metaverse or related technologies such as Virtual Reality (VR) and Augmented Reality (AR). Snowball sampling expanded the participant pool by having existing study participants refer additional respondents, ensuring a broader representation of perspectives within the targeted professional community.

### Data collection

The primary data for this study was collected through semi-structured interviews with 15 IT professionals in the Gambia. The interviews were conducted between April 15 and April 24, 2024. We obtained verbal informed consent from all participants prior to the interviews. The verbal consent process was necessary due to geographical limitations and was conducted online (Google Meet and Zoom). To ensure transparency, verbal consent was recorded in conjunction with the interviews. Participants were informed of their rights, including the voluntary nature of participation, confidentiality of their responses, and their right to withdraw from the study at any time.

The Institutional Review Board (IRB) of the Gambian government reviewed and approved this study, including the consent process. The IRB granted approval for the use of verbal consent in place of written consent due to logistical and geographical constraints. No waiver of consent was required. Data from these interviews are available at [https://doi.org/10.6084/m9.figshare.25659474].

In addition to interviews, the study incorporated a review of two key government documents: “The Gambia Digital Economy Diagnostic Report” and “The Gambia’s National Digital Economy Master Plan 2023-2033.” These documents provided a foundation for understanding The Gambia’s current digital governance context and its aspirations for digital transformation, informing the research.

The Table 2 provides an overview of the demographics of the participants, their backgrounds, and roles.

**Table 2:** Participant demographics, including their backgrounds and roles.

Interviewee Participant	Background	Role
Participant 1 (P1)	Information Technology Sector	IT Manager at UNDP, and Owner of Protect Company
Participant 2 (P2)	Data Scientist	CEO of Gomindz Academy
Participant 3 (P3)	Entrepreneur and Innovator	Founder and CEO of Kids in Technology, The Gambia
Participant 4 (P4)	Information Technology Sector	ICT Officer within the Central government
Participant 5 (P5)	Software Development	Software Engineer
Participant 6 (P6)	Education	Computer Science Lecturer, University of Gambia
Participant 7 (P7)	Data Scientist	Data Scientist and Project Researcher, Novia University of Applied Science
Participant 8 (P8)	Software Product Engineer	Head of Product Engineering and Quality Assurance, INSIST Global
Participant 9 (P9)	Software Development	Software Developer
Participant 10 (P10)	Software Development	Frontend Engineer
Participant 11 (P11)	Networking	Network Engineer, and Tutor
Participant 12 (P12)	Cyber Security	Cyber Security Specialist
Participant 13 (P13)	Software Development	Software Engineer
Participant 14 (P14)	Education	Computer Science Lecturer, University of Gambia
Participant 15 (P15)	Software Development	Software Developer

### Data analysis

To ensure a systematic and reliable data analysis process, we used NVivo software to methodically analyze the qualitative data collected from the interviews. The process started with the import of all interview transcripts into NVivo, which established a well-organized repository of all textual data. Subsequently, we utilized NVivo’s coding tools to segment the data into meaningful units. The codes were then generated, aligning with the themes that surfaced from the interviews, both deductively from the research questions and inductively as new themes emerged from the responses of the participants.

Following the coding process, we turned to NVivo’s node creation features to organize the codes into broader categories. This feature was particularly useful in reflecting common themes such as ‘digital literacy challenges’ and ‘infrastructure readiness,’ thus simplifying the analysis process. We also used NVivo’s matrix coding queries to compare themes between different groupings of participants, providing deeper insights into similarities and differences in perspectives.

Moreover, we employed NVivo’s query tools to explore relationships within the data, enabling us to identify co-occurring themes and draw conclusions about the challenges and opportunities of integrating Metaverse technologies into The Gambia’s digital governance. This comprehensive approach ensured a thorough and rigorous analysis of the qualitative data, which improved the reliability and depth of our findings.

Thematic analysis was employed to interpret the qualitative data collected from interviews with IT professionals in the Gambia. Initially, the recorded interviews were transcribed, allowing for a complete narrative of the participants’ insights. These transcripts were reviewed to recognize patterns and variations in responses. The next step was coding, where specific segments of the text were labeled to summarize the essence of the response [57]. This coding process was iterative, with initial codes refined and sometimes merged or subdivided as more data were analyzed.

NVivo software was used to manage these codes, tracking their application across different transcripts and facilitating the reorganization of codes into broader themes. The themes were then reviewed and refined to ensure that they accurately captured the key insights relevant to the research question. The study’s thematic analysis process revealed the key themes and patterns in the data, paving the way for a structured narrative on integrating Metaverse technologies into the Gambia’s digital governance.

### Validity, reliability, and ethical considerations

Validity in this qualitative study was achieved through systematic strategies. Purposive sampling targeted IT professionals whose experiences were directly relevant to the Gambia’s digital governance initiatives. This approach was balanced with diverse perspectives across various IT roles, enriching the findings by comparing interview data with government reports and existing literature.

The study’s insights, while rich for the context of the Gambia, may not directly apply to other regions. However, the qualitative data offer a valuable lens on the Metaverse’s integration into digital governance, useful for comparative analysis. Transparent documentation of research steps improves the credibility of the study in all contexts.

#### Ethical considerations

Ethical standards guided every stage of the research process, adhering to the principles outlined in the Declaration of Helsinki regarding research involving human participants [58]. We ensured that informed consent was obtained from all participants, highlighting the confidentiality of their responses and the voluntary nature of their participation. The ethical review and approval for this study were granted by the Gambian government Institutional Review Board that ensured adherence to the required standards of conduct. The study focused on non-personal and system-related information, maintaining participant privacy and anonymity.

### Limitations

Despite efforts to ensure a diverse range of perspectives through purposive and snowball sampling, reliance on interviews and specific government reports introduces biases. To counter this, the study incorporated data triangulation, systematically comparing interview insights with government documents and the existing literature, creating a balanced exploration of the topic.

The geographical distance between the researcher and the Gambia required online interviews, which presented challenges to the diversity of participants and the depth of the insights. To mitigate this, the interviews were structured to encourage open and detailed responses, with follow-up questions to probe deeper. Future research could extend the findings by employing broader sampling strategies, diversifying data sources, and exploring hybrid data collection methods.

## Findings

This section presents insights from interviews with IT professionals in The Gambia and a review of government reports. It focuses on integrating Metaverse technologies into the country’s digital governance, exploring key themes and implications.

### Perceived benefits and suitability of immersive technologies

After analyzing the interviews, the analysis revealed support for the potential of immersive technologies in the redesign of education, healthcare, public services, tourism, and infrastructure development.

#### Education and training reform

Many participants highlighted how VR and AR technologies could revolutionize education and training, making learning more interactive and engaging. For example, P1 noted the potential for immersive environments to reduce training costs by simulating realistic scenarios without the associated expenses. P3 emphasized that these technologies could make learning experiences more engaging, particularly in topics such as history, by immersing students in past events. P8 extended this perspective to healthcare, suggesting that VR and AR could support medical training and remote surgeries, thus enhancing professional competency.

Example quotes:

*So, sectors of governance that will add up mainly in this immersive environment, such as defense, will be a crucial area. They do training, and so on. And some of these trainings can sometimes be very expensive. (P1)*

*Yeah, so basically, these tools are emerging tools, and they are here with humanity to make life easier for us… It gives students a sense of reality. Let us think about history. When teaching kids history, they can see themselves in the history itself. (P3)*

*They can also help revolutionize education and training by providing interactive and engaging learning experiences that improve retention and understanding. (P6)*

#### Public service and governance enhancement

The participants pointed out the potential of immersive technologies to make public services more engaging and accessible, fostering a more transparent relationship between the government and its citizens. P6 discussed how VR and AR could improve service delivery by facilitating citizen engagement and transparency. P5 emphasized how these technologies could democratize political participation by making it more accessible and engaging to the public.

Example quotes:

*Public service can benefit from it. Digital governments can leverage technologies such as virtual reality and AR to enhance citizen engagement and transparent service delivery. (P6)*

*Because when it comes to politics in the Gambia, it is difficult to get the public involved… However, I believe that with augmented and virtual reality, people can participate and engage using those technologies. (P5)*

#### Infrastructure development and urban planning

Participants emphasized how VR and AR could transform planning processes, allowing enhanced visualization and stakeholder participation. P5 highlighted how immersive simulations could change planning from traditional methods to interactive simulations, involve stakeholders, and improve decision-making. Participants also noted the broader application of these technologies in infrastructural development and management, such as for ports, allowing a deeper understanding of proposed developments and facilitating real-time adjustments.

Example quotes:

*So, instead of just having those infrastructural developments on paper, how about we simulate them using virtual and augmented reality so that people who will benefit enjoy the feeling of having those things before actually having them. (P5)*

#### Cultural heritage and tourism enhancement

Immersive technologies were seen as a gateway to transform cultural heritage preservation and tourism experiences. P4 and P13 discussed how these technologies could create immersive experiences, allowing users to explore historical sites and cultural narratives, thus improving cultural preservation and tourism. This approach improves the appeal of the Gambia as a tourist destination and plays a crucial role in artistic education and preservation.

Example quotes:

*One of our primary sources of foreign currency is the tourism industry…so I think VR could greatly help virtual tourism. (P4)*

*So now, there can be an application, whether it be tourism or anything. In contrast, people can now visually go to a different place: we cannot visit every part of the country, but now people can visually be somewhere else. It may not feel genuine, but virtual reality in the future has that potential, whereas it can make people visit places at a low cost and with a low effort visually. (P13)*

### Challenges and concerns in adopting VR, AR, and Metaverse technologies

Integrating immersive technologies into the Gambia’s governance and public sectors presents several challenges and concerns:

#### Technical and infrastructure readiness

Participants highlighted concerns about The Gambia’s readiness to support immersive technologies. P1 emphasized the high cost of Internet access as a barrier, while P3 and P15 noted inconsistent electricity supply, affecting the operational feasibility of these technologies. Furthermore, P4 stressed the low levels of digital literacy in the population, highlighting the need to address these challenges to enable effective engagement with new technologies.

Example quotes:

*The first thing we need to consider when adopting immersive technology is the cost of the internet. (P1)*

*Yes, if we consider the infrastructure, it is also average. The internet connection here still needs to be fixed. You understand that it is slower than we expect. It is coupled with fluctuation of electricity. (P3)*

*One of the things that we need is stable amenities, which these high technologies or these new things need to perform. So, it seems risky to venture into them or to rely on them. So, we will probably need to clear the base and make it very readily available for electricity and the Internet. (15)*

#### Ethical and social considerations

Participants also raised concerns about privacy and data security within the Metaverse. P6 and P8 emphasized the need for robust privacy protections and cybersecurity measures to protect user information. P8 also pointed out the potential social detachment and psychological effects of prolonged immersion in virtual environments, emphasizing the importance of considering these factors.

Example quotes:

*One of my biggest concerns, honestly, is privacy and security. It will definitely be one of those big challenges and my concerns in terms of applications of the metaverse or any massive technologies. (P8)*

*But it can also detach most people from real-life conversations and socializations. Because we see people, some people feel more safe in the virtual world than in the real world, which should be of concern. (P13)*

#### Privacy and data protection in the Gambian Metaverse

As Metaverse technologies evolve and integrate into digital governance frameworks, robust privacy considerations are paramount. Most of the participants suggested that The Gambia should proactively develop a comprehensive legal framework that safeguards user data and ensures the sanctity of individual rights within these virtual environments. This necessitates extending existing Gambian privacy laws to encompass the novel forms of personal and biometric data generated in the Metaverse. In particular, a participant suggested that legislation must explicitly address the collection, processing, and sharing of personal information within virtual spaces, where the boundary between public and private interactions becomes increasingly blurred (P3). Drawing upon established international standards, such as the General Data Protection Regulation (GDPR), such a framework should mandate strict compliance requirements for all Metaverse platforms handling sensitive data.

As one interviewee said: *To really build trust with users and ensure that their privacy is protected on the Metaverse, we need to have clear accountability. That means things like making it mandatory for Metaverse providers to appoint Data Protection Officers. This will ensure a responsible and transparent handling of user data. By learning from what other countries are doing and tackling the unique challenges of the Metaverse head-on, The Gambia can create a safe and trustworthy space for everyone in this new digital world (P2).*

#### Security regulations

In order to safeguard the Gambian Metaverse, it is important to establish a comprehensive cybersecurity framework that addresses the security challenges posed by the convergence of virtual assets, personal data, and government services. Such a framework should enforce stringent encryption standards for all data exchanges within the Metaverse and mandate secure authentication methods, such as multifactor authentication, to prevent unauthorized access and protect user accounts.

As one interviewee put it: *In my view, it’s vital for The Gambia to establish a dedicated governmental body, maybe a task force, to oversee all these security measures in the Metaverse. This group should be responsible for developing emergency response plans in case of security breaches. We also need to make sure strong network security protocols are in place, especially to prevent things like DDoS attacks that could disrupt essential online services. By being proactive and tackling these security challenges head-on, The Gambia can create a secure and resilient Metaverse that protects both individuals and important digital infrastructure (P12).*

#### Digital asset management and intellectual property

It is imperative for The Gambia to establish a comprehensive legal framework to govern virtual economies within the Metaverse. This framework should address the creation, transfer and ownership of digital assets, as well as provide legal recognition and protection for intellectual property in digital spaces.

One interviewee argued that: *I think it is really smart to look at what other countries are already doing with this. You know, places like the US and Singapore have been dealing with some of these digital asset issues for a while now. We can learn a lot from their successes and avoid their mistakes when creating our own regulations. And with virtual money and tokens becoming so important in the Metaverse, we definitely need clear rules on how they are used. Things like taxes, preventing money laundering, and how smart contracts work. If we get the legal groundwork right for these new kinds of financial tool, it will encourage responsible innovation and boost our economy in the Metaverse. And that will make people feel more confident and secure in using them (P14).*

Ultimately, we believe that these measures will contribute to creating a secure and legally robust environment for economic activities within the Metaverse. By proactively addressing these challenges, The Gambia can nurture an innovative and thriving digital economy while upholding compliance with national and international financial laws, thereby ensuring the legitimacy and trustworthiness of the digital economy.

#### Policy and regulatory adjustments

The participants stressed the need for comprehensive regulatory frameworks to manage the complexities of virtual interactions and property rights effectively. The need for adaptive policies to address technological advancements was highlighted, ensuring a balance between innovation and protection.

The findings reveal a complex interaction between the potential benefits and challenges of integrating immersive technologies into the governance of Gambia. Although Metaverse offers distinctive opportunities in education, public services, infrastructure development, and tourism, these gains depend on addressing technical readiness, digital literacy, ethical considerations, and regulatory frameworks. These findings emphasize the need for a comprehensive, multi-stakeholder approach to successfully integrate Metaverse technologies into the Gambia’s digital governance.

## Discussion

The findings highlight both opportunities and challenges for integrating Metaverse technologies into the Gambia’s digital governance framework. This section investigates these findings, exploring key themes, potential challenges, and strategic recommendations, supported by the literature and participant insights.

### Key themes developed

The integration of immersive technologies presents an opportunity for the digital governance of Gambia, marking several vital themes that have the potential to improve sectors ranging from education to urban planning. Building upon insights from technology professionals and government documents such as “Gambia Digital Economy Master Plan 2023-2033” and “Digital Economy Diagnostic the Gambia” [27], we look at these themes that were created through insights from participants. They serve as pillars for exploring the impact of the Metaverse technology across various sectors. Let us examine each theme to understand its significance, challenges, and the way forward.

The Table 3 provides an overview of themes, associated codes, and example quotes related to the applications of Metaverse in various sectors.

**Table 3:** Themes, codes, and example quotes related to the Metaverse and its applications.

Themes	Codes	Example Quote
Metaverse in Educational and Training Reform	Immersive Educational Experiences, Defense Training, Educational System Integration, Revolutionizing Education, Enhanced Learning and Engagement, Educational Simulations, Healthcare Integration, Healthcare and Medical Training, Health Sector Enhancement, Road safety training simulations, Innovative Healthcare Applications, Industrial Training Enhancement	“So, the sectors of governance that will add up mostly in this immersive environment, such as defense, will be one crucial area. They do much training and so on. Moreover, some of these trainings sometimes can be very costly”. P1
Public Service and Governance Enhancement	Enhanced Public Service, Diplomatic Training Simulations, Improved Government-Citizen Interaction, Remote Government Collaboration	“Public service can benefit from it. Digital governments can leverage technologies like VR and AR to enhance citizen engagement and transparent service delivery.” P6
Digital Infrastructure and Urban Development	Infrastructure Development Visualization, Urban Planning Visualization, Port Authority Virtualization	“One of the things I believe it can focus on is planning, such as infrastructure planning. Because it is like, instead of building a whole town in and of itself, it can be built into the digital space.” P14
Cultural Heritage and Tourism Enhancement	Virtual Tourism Potential, Cultural Sectors, Virtual Tourism, Entertainment Industry Potential	“One of our primary sources of foreign currency is the tourism industry…so I think VR could help a lot in virtual tourism.” P4

#### Education and training reform

The transformative potential of VR and AR in education and training emerged as a key theme. These technologies can create dynamic, interactive learning experiences that improve knowledge retention and skill development across sectors. P6 emphasized how VR and AR could revolutionize education by providing interactive and engaging learning experiences that improve retention and understanding. In healthcare, VR simulations can provide immersive training for medical professionals, improving their skills and confidence in complex procedures [49]. P8 also emphasized the potential for VR and AR to support medical training and remote surgeries, highlighting how these technologies can contribute to professional competency.

#### Public service and governance enhancement

The Metaverse offers opportunities for public service enhancement, streamlining service delivery, and fostering transparency and engagement. Virtual platforms, such as digital consular services, can improve accessibility and responsiveness in areas such as tax consultations and healthcare appointments [48]. P6 discussed how VR and AR could improve service delivery by facilitating citizen engagement and transparency. The potential of VR and AR to facilitate diplomatic training and political participation suggests new avenues for citizen participation and collaboration. P5 emphasized how these technologies could democratize political participation by making it more accessible and engaging to the public.

#### Infrastructure development and tourism transformation

VR and AR technologies provide opportunities for the development of infrastructure and urban planning, enabling comprehensive visualization and simulation of projects before implementation. This participation facilitates feedback and adjustments, ensuring optimal outcomes. P5 highlighted how immersive simulations could change planning from traditional methods to interactive simulations, involve stakeholders, and improve decision-making. In addition, in tourism, immersive experiences offer innovative ways of engaging visitors, enhancing cultural heritage preservation, and expanding educational opportunities [50]. P4 and P13 discussed how these technologies could create immersive experiences, allowing users to explore historical sites and cultural narratives, enhancing both tourism and cultural preservation.

### Bridging the digital divide: A multi-tiered approach to digital literacy in The Gambia

To effectively integrate Metaverse technologies and other digital advances into Gambian society, the majority of participants suggested that a comprehensive and multi-tiered approach to digital literacy is essential. This strategy should encompass the following key components:

#### Digital literacy for students

Integrating digital literacy into the national curriculum at both the primary and secondary levels is crucial to prepare future generations. This should include mandatory courses focusing on fundamental ICT skills, Internet safety, and practical virtual communication tools (P6). Existing initiatives, such as the Progressive Science Initiative and Progressive Math Initiative (PSI-PMI), can be expanded to incorporate digital literacy components. Using computer-assisted learning techniques within these programs will further enhance their effectiveness and ensure that students are well equipped to navigate the digital landscape (P14).

#### Civil servant training programs

Equipping civil servants with the necessary digital skills is vital to a smooth transition to Metaverse and other digital platforms in the delivery of public services. Designed digital literacy programs for civil servants should prioritize comprehensive and up-to-date training sessions on the use of virtual tools, the practice of secure data handling (P12), and the effective integration of technology into various aspects of the public service (P7). Collaborations with international agencies and private sector organizations can provide valuable support through resource sharing and expertise, ensuring that these programs are comprehensive and up-to-date (P1).

#### Communty-based digital literacy initiatives

Community-driven digital literacy programs are essential to ensure inclusivity and bridge the digital divide, particularly in rural areas with limited access to technology (P4). Local ICT centers can play a crucial role by offering free workshops on using digital devices. In addition, mobile digital literacy units can be deployed to reach underserved regions, extending the benefits of these programs to all citizens (P3). Public-private partnerships and collaborations with international development organizations can provide crucial support and resources for these community-based initiatives (P8).

By implementing this multifaceted strategy, The Gambia can cultivate a digitally literate population, empower individuals, strengthen public services, and foster a thriving digital economy.

### Challenges and other recommendations

#### Technical readiness

The Gambia’s digital infrastructure must be robust enough to support immersive technologies, necessitating investments in broadband connectivity and consistent internet access. *The Gambia Digital Economy Diagnostic* report by Muller et al. [27] highlights the need for significant improvements in Internet connectivity and digital infrastructure to support digital governance ambitions. In addition, expanding digital literacy programs can equip citizens with the necessary skills to navigate and benefit from virtual environments, reducing the digital divide. P4 emphasized the low levels of digital literacy in The Gambia, highlighting the need for targeted digital literacy programs.

#### Ethical and privacy considerations for Metaverse integration

As The Gambia begins to incorporate Metaverse technologies into its digital governance framework, and it is crucial to consider the ethical and privacy implications carefully. The immersive nature of the Metaverse allows for the collection of highly sensitive personal information, including biometric data, which gives rise to significant privacy concerns.

The General Data Protection Regulation (GDPR) [59] offers a strong foundation to regulate personal data in the Metaverse. The core principles of GDPR: (i) ensuring user consent, (ii) limiting data collection to what is strictly necessary, and (iii) guaranteeing user rights over their data, are particularly relevant in the context of virtual reality, where sensitive personal information, including biometric data, can be collected. Without implementing provisions similar to those in the GDPR, The Gambia could be at risk of privacy breaches. By adopting GDPR-like provisions, The Gambia would be able to protect the privacy of citizens and ensure that Metaverse platforms operate with transparency and accountability. By proactively protecting the privacy of its citizens within Metaverse, The Gambia can mitigate these risks. This involves establishing clear guidelines on data ownership, user rights, and the responsibilities of Metaverse platform providers with respect to data security and transparency.

To provide a robust framework for privacy in the Metaverse, The Gambia can take lessons from countries that have successfully implemented comprehensive digital privacy laws. Estonia, for example, is recognized as a leader in e-Governance, with strong privacy laws supporting its national digital identity system [5]. The country’s X-Road platform ensures secure data exchange between citizens and public services, backed by stringent privacy protections. Similarly, Singapore’s Personal Data Protection Act (PDPA) balances the need for innovation in digital services with strong safeguards for personal data [13]. These countries demonstrate how privacy regulations can coexist with digital innovation and their experiences offer valuable insights for The Gambia. By tailoring aspects of these frameworks to fit its own context, The Gambia can develop a regulatory environment that fosters trust and security in the Metaverse.

In the Gambian context, the implementation of GDPR-like privacy regulations must consider the local infrastructure and legal limitations. Although the principles of user consent, transparency, and data minimization are universally applicable, they must be adapted to the specific challenges faced by The Gambia, such as limited digital literacy and uneven internet access. A phased approach to regulation, starting with awareness campaigns and capacity building for stakeholders, would ensure the smooth adoption of these standards. Furthermore, the establishment of a regulatory body to oversee privacy compliance in digital environments would be crucial to ensuring that Metaverse platforms are held accountable for the protection of user data.

Implementing such a framework will not only safeguard individual privacy, but also promote trust and accountability within the Metaverse ecosystem. As policymakers, legal experts, and technology stakeholders, your role in this process is crucial. Your actions will facilitate the responsible development and integration of the Metaverse into The Gambia’s digital landscape, empowering you to shape the future of privacy in the Metaverse.

#### Ethical and social considerations

Privacy and security concerns need to be addressed to maintain trust in Metaverse technologies. Comprehensive cybersecurity measures and privacy protections are essential for safeguarding user information [51]. P8 stressed the need for robust privacy protections and cybersecurity measures, emphasizing how these technologies introduce significant privacy and security concerns. Furthermore, the potential for social detachment and psychological impacts of prolonged immersion requires ethical guidelines to ensure balanced digital experiences.

#### Policy and regulatory adjustments

The integration of Metaverse technologies into The Gambia’s digital governance framework necessitates comprehensive regulatory frameworks to manage the complexities of virtual interactions and property rights. Robust data protection and privacy measures are essential to protect user information. The General Data Protection Regulation (GDPR) of the European Union offers valuable lessons on how adaptive policies can balance innovation and protection, ensuring secure digital interactions within the Metaverse [52]. In addition, frameworks must address virtual property rights to ensure fair transactions and protect users, recognize digital assets, and manage ownership transfer and disputes effectively.

To foster inclusive governance, policies should also support digital literacy programs, equipping citizens with the skills necessary to navigate and engage with Metaverse technologies. By addressing these areas, regulatory frameworks can ensure that the integration of Metaverse technologies into the Gambia’s digital governance framework is secure, fair, and accessible to all societal segments.

### Multi-stakeholder collaboration and future research

Achieving successful integration of the Metaverse into The Gambia’s governance framework requires collaboration among various stakeholders. *The Digital Economy Master Plan 2023-2033 emphasizes* multistakeholder collaboration, highlighting the need for partnerships between the government, the private sector, and international allies. P1 also emphasized the importance of collaboration, stressing the need for comprehensive strategies to address technical, ethical, and regulatory challenges, ensuring inclusivity and innovation in digital governance.

This study opens several avenues for future research. The next step involves broadening participant diversity to include a broader range of stakeholders from various sectors, enhancing the findings’ applicability and depth. Future studies could benefit from a mixed method approach, combining qualitative insights with quantitative data, to offer a more rounded perspective on the adoption and impact of Metaverse technologies in governance.

Furthermore, comparative research in different geopolitical contexts could shed light on universal challenges and unique strategies, providing a global view of the evolution of digital governance. Looking deeper into the ethical, social, and political dimensions of the Metaverse will also be crucial, particularly in addressing privacy concerns, equity, and the potential for digital divides. Lastly, a longitudinal approach, observing the Metaverse’s role in governance over time, could capture the patterns of technological adoption and its long-term effectiveness. By pursuing these avenues, future research can build on this study’s foundation, contributing to an understanding of the Metaverse’s potential to revolutionize digital governance worldwide.

**Fig 4:**
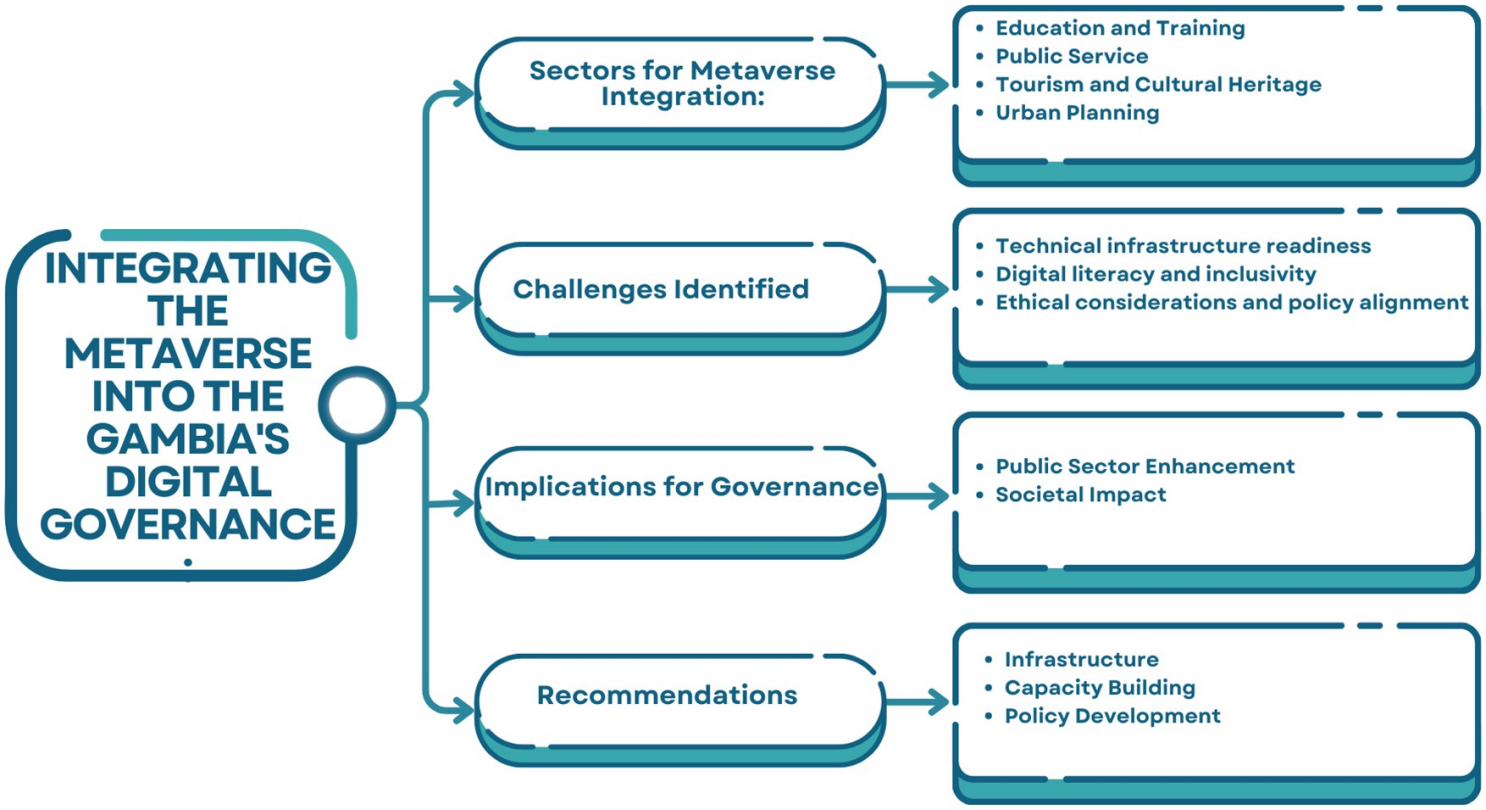
Concept map for the integration of immersive technologies into The Gambia’s digital governance.

Fig 4 presents an overview of the integration of Metaverse technologies into the digital governance of The Gambia. It outlines key sectors for integration, such as education, public services, tourism, and urban planning. The challenges identified include technical infrastructure readiness, gaps in digital literacy, and ethical considerations. The implications for governance are framed in terms of improving the public sector and social impact. To address these challenges, the recommendations include focusing on infrastructure development, capacity building, and policy formulation to support effective adoption.

## Conclusion

This study has explored the use of Metaverse technologies, such as Virtual Reality (VR) and Augmented Reality (AR), in the Gambia’s approach to digital governance. The study shows that these technologies could significantly improve the way government services are delivered and increase public participation in government processes. Sectors like education, healthcare, public services, and tourism could become more effective and accessible, benefiting from the immersive and interactive capabilities of these technologies.

However, there are significant challenges to overcome before these benefits can be realized. The Gambia needs to improve its digital infrastructure, increase the digital skills of its population, and establish supportive laws and policies. Challenges such as unreliable Internet and electricity, high technology costs, and low digital literacy rates require urgent attention to make these technologies workable. Future research should involve more stakeholders, seek affordable technology solutions that are suitable for the economic situation of Gambia, and study the long-term effects of these technologies. Comparing the progress of Gambia with that of other countries could also provide valuable lessons and insights.

Although introducing Metaverse technologies into the Gambia governance system presents various obstacles, it also offers considerable opportunities to modernize and improve how government services are provided and how citizens engage with their government. With careful planning and ongoing efforts to address technological and social challenges, Gambia has the potential to leverage these advanced technologies to improve governance and delivery of public services.
